# Metabolic Enzymes in Viral Infection and Host Innate Immunity

**DOI:** 10.3390/v16010035

**Published:** 2023-12-24

**Authors:** Chao Qin, Taolin Xie, Wayne Wei Yeh, Ali Can Savas, Pinghui Feng

**Affiliations:** Section of Infection and Immunity, Herman Ostrow School of Dentistry, Norris Comprehensive Cancer Center, University of Southern California, Los Angeles, CA 90089, USA

**Keywords:** metabolic enzymes, cell metabolism, viral infection, innate immunity, interferon, inflammatory response, antiviral therapy

## Abstract

Metabolic enzymes are central players for cell metabolism and cell proliferation. These enzymes perform distinct functions in various cellular processes, such as cell metabolism and immune defense. Because viral infections inevitably trigger host immune activation, viruses have evolved diverse strategies to blunt or exploit the host immune response to enable viral replication. Meanwhile, viruses hijack key cellular metabolic enzymes to reprogram metabolism, which generates the necessary biomolecules for viral replication. An emerging theme arising from the metabolic studies of viral infection is that metabolic enzymes are key players of immune response and, conversely, immune components regulate cellular metabolism, revealing unexpected communication between these two fundamental processes that are otherwise disjointed. This review aims to summarize our present comprehension of the involvement of metabolic enzymes in viral infections and host immunity and to provide insights for potential antiviral therapy targeting metabolic enzymes.

## 1. Introduction

Metabolic enzymes are indispensable for cell survival and the maintenance of cellular homeostasis. They play critical roles across a wide spectrum of metabolic pathways due to their distinct enzymatic activity, e.g., carboxylases, dehydrogenases, lipoxygenases, oxidoreductases, kinases, lyases, and transferases. As obligate intracellular pathogens, viruses rely on host cell machinery to power the biosynthesis of various components essential for progeny production, such as nucleic acids, proteins, and lipids. Therefore, it is not unexpected that viruses reprogram cellular metabolism to maximize progeny virion production. To do this, viruses evolved multiple strategies to hijack cellular metabolic enzymes. Viruses can increase the expression of metabolic enzymes or promote their activation. These metabolic enzymes serve essential roles in their replication: they increase the production and thus availability of essential macromolecules that are utilized to facilitate the specific stages of the viral life cycle. A few viruses, particularly herpesviruses, encode their own metabolic enzymes. These viral enzymes often catalyze the rate-limiting steps of nucleotide biosynthesis, thereby unleashing host cell restrictions to promote viral replication. This adaptation highlights the virus’s intricate strategy that co-opts cellular resources to benefit viral infection, ultimately promoting its successful replication and consequent dissemination within the host. Uncovering the mechanisms by which viruses hijack cellular metabolic enzymes or employ their own enzymes to enhance replication represents an effective avenue to identify potential targets for antiviral interventions.

Cellular metabolism plays a pivotal role within the immune system as well. Metabolic processes are essential for providing precursors and meeting the unique energy requirements associated with various immunological processes. Innate immunity, one of the integral branches of the immune system, serves as the first line of host defense against invading pathogens. Within the intricate metabolic networks associated with immune activation, numerous enzymes are intrinsically capable of modulating host immunity. Under certain circumstances, these enzymes catalyze the rate-limiting steps and thus govern the nutrient flow through the pathways necessary to fulfill the specific energetic or metabolic needs of the immune response. Alternatively, key metabolic enzymes may regulate the synthesis or consumption of metabolites that directly participate in immune signaling events. Furthermore, certain metabolic enzymes have evolved to demonstrate unique enzymatic activity in the immune response, which is distinct from their conventional enzymatic activities.

In this review, we delve into the essential metabolic enzymes implicated in viral infections and their critical roles in immune regulation. We highlight mechanistic insights and explore potential opportunities for clinical interventions. Given the broad topics that we are going to summarize, we will only focus on metabolic enzymes involved in glycolysis, the TCA cycle, nucleotide synthesis, and lipogenesis. Additionally, various virus types will be included, such as DNA viruses (e.g., herpesvirus, hepadnavirus, adenovirus, and papovavirus), RNA viruses (e.g., flavivirus, coronavirus, and picornavirus), and retroviruses.

## 2. Metabolic Enzymes and Viral Infection

### 2.1. Metabolic Enzymes in Viral Life Cycle

#### 2.1.1. Metabolic Receptor-Mediated Viral Entry

**Figure 1:**
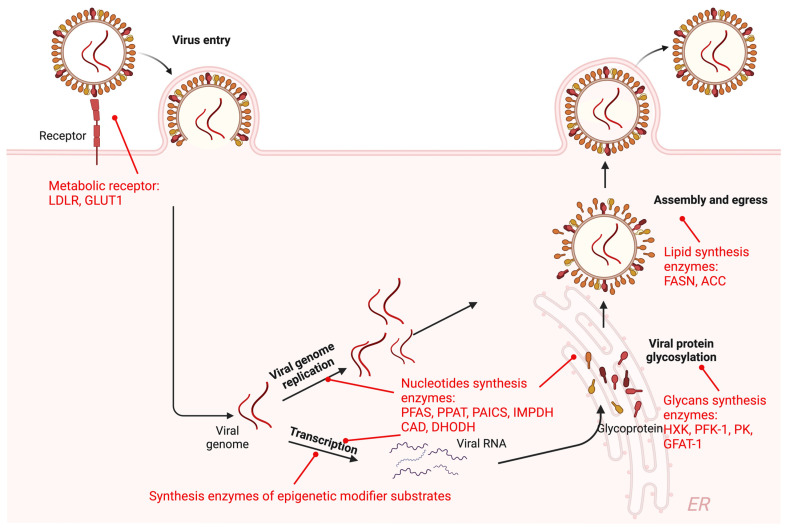
Metabolic enzymes in viral life cycles. Figure shows a schematic illustration of viral life cycles including entry, viral genome replication, transcription, viral protein maturation, assembly, and egress. Metabolic enzymes involved in the viral life cycle are highlighted in red. Abbreviations: GLUT, glucose transporter; LDLR, low-density lipoprotein receptor; CAD, carbamoyl-phosphate synthetase, aspartate transcarbamoylase, and dihydroorotase; DHODH, dihydroorotate dehydrogenase; PPAT, phosphoribosyl pyrophosphate amido transferase; PFAS, phosphoribosylformylglycinamidine synthase; PAICS, phosphoribosylaminoimidazole carboxylase and phosphoribosylamino-imidazolesuccinocarboxamide synthase; IMPDH, inosine monophosphate dehydrogenase; ACC, acetyl-CoA carboxylase; FASN, fatty acid synthase; HXK, hexokinase; PFK-1, phosphofructokinase-1; PK, pyruvate kinase; GFAT-1, glutamine fructose-6-phosphate amidotransferase 1. Created with Biorender.com.

Viruses’ entry into cells is initiated by their attachment to receptors and is followed by an internalization process [1,2] (Figure 1). Two main internalization routes enable the entry of viruses into the cell: the endocytic and non-endocytic pathways. Interestingly, receptors for nutrient uptake are frequently exploited by viruses to mediate their entry. For instance, glucose transporter 1 (GLUT1) can serve as a receptor for human T-lymphotropic virus type 1 (HTLV-1) to attach to target cells [3]. Human rhinovirus 2 undergoes receptor-mediated endocytosis after interaction with a low-density lipoprotein receptor (LDLR) [4]. Hepatitis C virus (HCV) utilizes LDLR to gain entrance into hepatocytes, but the exact mechanisms remain unknown [5]. Human folate receptor-α is one of the attachment factors for both Ebola virus (EBOV) and Marburg virus [6,7]. It will not be surprising that more receptors akin to the above-mentioned ones will be characterized to mediate viral entry. Adding an intriguing dimension, metabolic enzymes can also act as suppressors in the context of viral entry. It was reported that cholesterol-25-hydroxylase (Ch25h) can convert cholesterol to a soluble antiviral factor, 25-hydroxycholesterol (25HC) [8]. 25HC inhibits viral entry by blocking membrane fusion between viruses and cells [8]. Strikingly, Ch25h expression or 25HC treatment is capable of inhibiting various viruses, including vesicular stomatitis virus (VSV), herpes simplex virus (HSV), human immunodeficiency virus (HIV), murine gammaherpesvirus 68 (MHV68), Ebola virus (EBOV), Rift Valley fever virus (RVFV), Russian spring–summer encephalitis virus (RSSEV), Nipah viruses, and severe fever with thrombocytopenia syndrome virus (SFTSV) [8,9].

#### 2.1.2. Metabolic Enzymes in Viral Transcription and Replication

Metabolic enzymes are also active players in viral gene expression and replication (Figure 1). In the case of DNA viruses and retroviruses, their genomes can form episomes and integrate into the host chromosome, respectively. The herpesvirus genomes are maintained as circular genomes assembled with histones in latently infected cells. The similarity in the genetic composition and structure between viruses and the host cell allows viral gene transcription to be regulated by DNA and histone modifications, akin to what occurs in eukaryotic cells. By manipulating epigenetic regulation, viruses can avoid epigenetically repressed gene expression by host cells [10]. For viruses exhibiting persistent infection like human cytomegalovirus, KSHV, EBV, HSV-1, and HIV, epigenetic regulation is used to control the switch between latent and lytic phases [11,12,13,14,15]. Epigenetic regulatory enzymes, such as histone demethylases like lysine-specific demethylase 1 (LSD1) and members of the Jumonji domain 2 (JMJD2) family, histone methyltransferase Set1, and lysine methyltransferase 2A (MLL1), are essential for virus replication, including but not limited to influenza A virus, HIV, KSHV, HSV, and varicella zoster virus [16,17,18,19]. The activity of these enzymes is intimately regulated by the levels of their substrates [20], such as acetyl-coenzyme A (acetyl-CoA), S-adenosylmethionine (SAM), α-ketoglutarate (α-KG), and nicotinamide adenine dinucleotide (NAD) [21,22,23]. Furthermore, metabolic enzymes can catalyze post-translational modifications of key viral players, such as transcription factors, leading to altered viral gene expression [24,25,26]. For instance, phosphoribosylformylglycinamidine synthetase (PFAS) was reported to deamidate replication transactivator (RTA) of Kaposi’s sarcoma-associated herpesvirus (KSHV), which impairs the binding of RTA to the importin complex responsible for nuclear import, thus diminishing RTA nuclear localization and transcriptional activation [24]. Furthermore, all gamma herpesvirus RTA homologues appear to be deamidated by PFAS, suggesting a common mechanism by which viral replication is intimately coupled to the activity of a nucleotide-synthetic enzyme. PFAS acts as a scaffold in the assembly of the so-called purinosome in de novo purine synthesis, raising the interesting question of how a cell’s metabolic status affects KSHV replication status.

#### 2.1.3. Metabolic Enzymes in Mature Virion Production

Viruses enclose their viral genomic material within capsid proteins to protect it from environmental damage and enable its safe delivery into host cells [27]. Embedded in the envelope, glycoproteins mediate viral entry and contribute to the assembly and egress of progeny virions during the late stages of infection [28] (Figure 1). Immediately after translation, these naked proteins undergo the addition and modification of glycans, a process that occurs in the endoplasmic reticulum (ER) and Golgi apparatus. Viruses utilize the cellular glycosylation pathway to mature their glycoproteins, and this modification is essential for proper protein folding and function [29]. The availability of glycans for glycosylation depends on metabolic pathways, including the glycolysis, nucleotide, and hexosamine biosynthetic pathway. The enzymes involved in glycan synthesis are crucial for glycosylation [30], including the rate-limiting enzymes hexokinases, phosphofructokinase-1, and pyruvate kinases for glycolysis; multiple enzymes for UTP synthesis; and glutamine-fructose-6P amidotransferases for the hexosamine biosynthetic pathway [31,32]. Glycosyltransferases use activated glycans as their sugar donors [33]. The activation of glycans requires the addition of a nucleotide to a monosaccharide, forming a nucleotidyl sugar. Among all nucleotide sugars, UDP-sugars are particularly important precursors for protein glycosylation [34]. UDP-glucose analogs have shown strong antiviral activity against diverse viruses, including herpes simplex virus type 1 (HSV-1), adenovirus type 5, vaccinia virus (VACV), poliovirus type 1, encephalomyocarditis virus, vesicular stomatitis virus, influenza virus, and measles virus [35]. The generation of UDP-sugars requires not only nucleotides and glycans as substrates but also enzymes like pyrophosphorylases that use UTP as a source or uridyltransferases to add UDP to the sugars [34]. Targeting nucleotide sugar synthesis can significantly reduce the production of infectious virions, such as HIV, human cytomegalovirus (HCMV), influenza A virus, and vesicular stomatitis virus [36,37].

Lipids, as the major components of the virion envelope, are crucial for virus packaging and egress from the infected cells [38] (Figure 1). Inhibitors that target key enzymes of lipid synthesis can significantly impair the production of infectious virions [39,40,41,42,43]. For instance, the fatty acid synthase inhibitor Orlistat, long-chain acyl-CoA synthetase inhibitor Triacsin C, and cholesterol synthesis inhibitors statins all demonstrate apparent antiviral activity against SARS-CoV-2, dengue virus and Zika virus [44,45]. However, it remains unknown whether these inhibitors will have side effects on bystander cells in animal models and humans. The safety profile of statins in humans may alleviate some of the concern when considering these drugs as antiviral agents.

### 2.2. Viruses Hijack Cellular Metabolic Enzymes to Reprogram Cell Metabolism

#### 2.2.1. Glycolytic Enzymes

**Figure 2:**
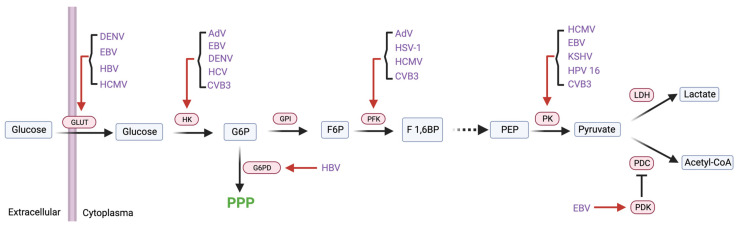
Glycolytic enzymes are hijacked by viruses. Figure shows a schematic illustration of the cross-regulation between viruses and glycolytic enzymes. Abbreviations: GLUT, glucose transporter; HK, hexokinase; GPI, glucose-6-phosphate isomerase; PK, pyruvate kinase; PFK, phosphofructokinase; LDH, lactate dehydrogenase; PDC, pyruvate dehydrogenase complex; PDK, pyruvate dehydrogenase kinase; G6PD, glucose-6-phosphate dehydrogenase; G6P, glucose-6 phosphate; F6P, fructose 6-phosphate; F 1,6BP, fructose-1,6-bisphosphate; PEP, phosphoenolpyruvic acid; PPP, pentose phosphate pathway; DENV, dengue viruses; EBV, Epstein–Barr virus; HBV, hepatitis B virus; HCMV, human cytomegalovirus; AdV, adenoviruses; HCV, hepatitis C virus; CVB3, coxsackievirus B3; HSV-1, herpes simplex virus type 1; HPV 16, human papillomavirus type 16; KSHV, Kaposi’s sarcoma herpesvirus. Created with Biorender.com.

Glycolysis is the first step in the breakdown of glucose to extract energy for cellular metabolism. Different viruses were shown to employ distinct mechanisms to reprogram glycolysis (Figure 2) (Table 1). The adenovirus ORF E4 binds MYC in the nucleus to enhance MYC’s transcriptional activation of key glycolic enzymes, such as hexokinase 2 (HK2) and phosphofructokinase-muscle type (PFKM) [46]. In addition, an adenovirus 13S-encoded E1A isoform up-regulates the expression of glycolytic enzymes to fuel aerobic glycolysis [47]. Herpesviruses are also capable of tuning glycolysis. HSV-1 was reported to increase the expression and activity of phosphofructokinase (PFK-1) to promote glucose consumption and glycolytic metabolite production [48]. HCMV infection in human fibroblasts enhances the transcription of several glycolytic enzymes (e.g., PFK, pyruvate kinase) and promotes PFK activity, resulting in an increased glycolytic flux [49]. The major immediate–early protein IE72 of HCMV down-regulates the GLUT1 receptor, while enhancing the expression of GLUT4 at the mRNA and protein levels [50]. Notably, GLUT4 exhibits a three-fold higher affinity for glucose than GLUT1. Treatment with indinavir, a drug that selectively inhibits the GLUT4 receptor, effectively reduces both glucose uptake and HCMV replication [50]. EBV-encoded latent membrane protein 1 (LMP1) stabilizes MYC to promote transcription of HK2 [51]. The up-regulation of HK2 is responsible for EBV-increased glycolysis and correlates with the poor overall survival of nasopharyngeal carcinoma (NPC) that is caused by EBV infection in patients [51]. LMP1 also promotes the mRNA expression and stabilizes the protein of GLUT1, thus potently increasing GLUT1 receptor activity [52,53]. Furthermore, LMP1 overexpression promotes the transcription of hypoxia-inducible factor-1α (HIF-1α), which further enhances the expression of pyruvate kinase muscle isozyme M2 (PKM2) and pyruvate dehydrogenase kinase 1 (PDK1) [54,55]. In KSHV-infected cells, the inhibition of PKM2 impedes aerobic glycolysis [56]. Notably, KSHV demonstrates the capability to up-regulate PKM2 [56]. Moreover, the inhibition of PKM2 also diminishes endothelial cell migration and differentiation, along with reducing the angiogenic potential of KSHV-infected cells [56]. Dengue virus (DENV) was reported to up-regulate the expression of GLUT1 and HK2, which sharply increases aerobic glycolysis [57]. HPV type 16 E7 interacts with PKM2, inducing its transition to a dimeric state that diminishes PKM2′s affinity for PEP in the final phase of glycolysis. This interaction may serve as a mechanism to redirect glycolytic intermediates for anabolic metabolism, simultaneously compensating for the reduced energy production due to augmented glutamine metabolism [58,59]. Hepatitis virus employs multiple strategies to activate glycolysis. Pre-S2 mutant protein of HBV activates the mTOR signaling cascade to promote GLUT1 translocation and aerobic glycolysis [60]. HBV up-regulates the G6PD expression driven by the X protein-mediated activation of nuclear factor erythroid 2–related factor 2 (Nrf2) [61]. The HCV NS5A protein interacts with HK2 and increases its activity, which contributes to HCV infection-induced glycolysis [62]. Coxsackievirus B3 (CVB3) increases the expression of HK2, PFKM, and PKM2, and their inhibitors significantly impede CVB3 replication [63]. The ongoing pandemic SARS-CoV-2 increases glycolysis to fuel its replication in monocytes through an HIF-1α-dependent pathway, while the treatment of cells with 2-Deoxy-D-glucose (2-DG), an HK inhibitor, efficiently blocked the replication of SARS-CoV-2 [64]. These studies provide an overview into the detailed interaction between viral pathogens and the glycolytic pathway, offering mechanistic insights into viral glycolytic reprogramming and exposing key enzymes for antiviral interventions.

#### 2.2.2. Enzymes in Glutamine Metabolism and TCA Cycle

**Figure 3:**
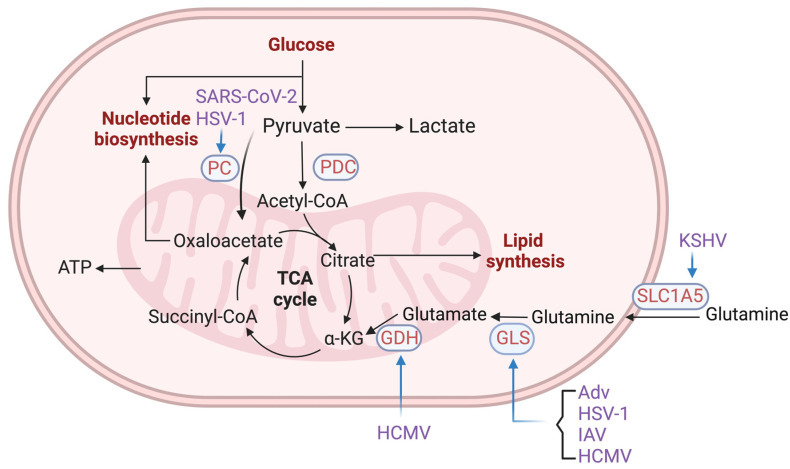
Enzymes in TCA cycle are hijacked by viruses. Figure shows a schematic illustration of the cross-regulation between viruses and TCA-cycle enzymes. Abbreviations: PC, pyruvate carboxylase; PDC, pyruvate dehydrogenase complex; GDH, glutamate dehydrogenase; GLS, glutaminase; SLC1A5, solute carrier family 1 member 5; α-KG, α-ketoglutarate; ATP, adenosine triphosphate; SARS-CoV-2, severe acute respiratory syndrome coronavirus 2; HCMV, human cytomegalovirus; AdV, adenoviruses; HSV-1, herpes simplex virus type 1; KSHV, Kaposi’s sarcoma herpesvirus; IAV, influenza A virus. Created with Biorender.com.

In addition to glucose, glutamine serves as another major source of nitrogen and carbon. The tricarboxylic acid (TCA) cycle plays a pivotal role in both ATP production and the synthesis of biomolecules essential for viral replication (Figure 3) (Table 1). Adenovirus activates MYC to promote glutamine uptake and usage in the reductive carboxylation that converts α-ketoglutarate (α-KG) to citrate/isocitrate [65]. KSHV infection in endothelial cells induces the expression of the glutamine transporter SLC1A5, thereby increasing glutamine uptake and intracellular glutamine levels. Inhibition of SLC1A5 or glutaminase (GLS), which catalyzes glutamine hydrolysis, induces apoptosis in KSHV latently infected cells, which can be rescued by α-KG [66]. GLS inhibition was also shown to impede the replication of adenovirus, HSV-1, and influenza A virus (IAV) in human primary cells [65]. The depletion of pyruvate carboxylase (PC) and aspartate transaminase 2 (GOT2) reduces HSV-1 replication, which is likely caused by the reduced synthesis of aspartate, which is crucial for de novo pyrimidine synthesis [67]. HCMV infection elevates glutamine utilization by increasing the expression and activity of GLS and glutamate dehydrogenase (GDH), which are crucial for its replication [68]. Similarly, HCV was reported to increase the transcript levels of key enzymes of glutamine metabolism in cultured cells and in liver biopsies of chronic HCV patients [69], whereas SARS-CoV-2 enhances the entry of glucose carbon into the TCA cycle by up-regulating the PC expression [70]. Meanwhile, SARS-CoV-2 decreases oxidative glutamine metabolism, while preserving reductive carboxylation [70], which likely directs glutamine carbon to support lipid synthesis, which is essential for the replication of enveloped viruses.

#### 2.2.3. Enzymes of Nucleotide Synthesis

**Figure 4:**
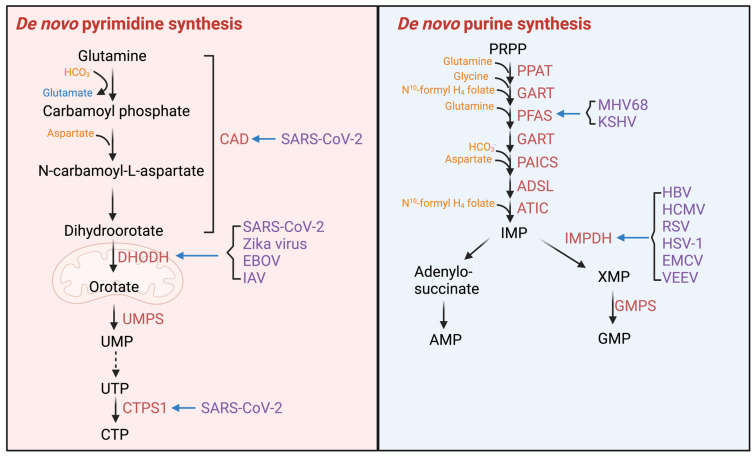
Enzymes in nucleotide synthesis are hijacked by viruses. Figure shows a schematic illustration of the cross−regulation between viruses and nucleotide synthesis enzymes. Abbreviations: CAD, carbamoyl−phosphate synthetase, aspartate transcarbamoylase, and dihydroorotase; DHODH, dihydroorotate dehydrogenase; UMPS, uridine monophosphate synthase; UMP, uridine monophosphate; UTP, uridine−5′-triphosphate; CTPS1, cytidine triphosphate synthase 1; CTP, cytidine 5′−triphosphate; SARS−CoV−2, severe acute respiratory syndrome coronavirus 2; EBOV, Ebola virus; IAV, influenza A virus; PRPP, phosphoribosyl diphosphate; PPAT, phosphoribosyl pyrophosphate amido transferase; GART, glycinamide ribonucleotide transformylase; PFAS, phosphoribosylformylglycinamidine synthase; PAICS, phosphoribosylaminoimidazole carboxylase and phosphoribosylamino−imidazolesuccinocarboxamide synthase; ADSL, adenylosuccinate lyase; ATIC, 5−aminoimidazole−4−carboxamide ribonucleotide formyltransferase; IMPDH, inosine monophosphate dehydrogenase; GMPS, guanine monophosphate synthase; IMP, inosine monophosphate; AMP, adenosine monophosphate; XMP, xanthosine monophosphate; GMP, guanosine monophosphate; MHV68, murine gammaherpesvirus 68; HBV, hepatitis B virus; HCMV, human cytomegalovirus; HSV−1, herpes simplex virus type 1; KSHV, Kaposi’s sarcoma herpesvirus; RSV, respiratory syncytial virus; EMCV, encephalomyocarditis virus; VEEV; Venezuelan equine encephalitis virus. Created with Biorender.com.

Nucleotide synthesis produces essential materials for the viral productive infection cycle, and viruses fine-tune nucleotide metabolism (Figure 4) (Table 1). SARS-CoV-2 was reported to activate carbamoyl-phosphate synthetase, aspartate transcarbamoylase, and dihydroorotase (CAD), and thus de novo pyrimidine synthesis by Nsp9 [71]. Activated CAD also deamidates RelA, which shuts down NF-κB activation and the expression of inflammatory genes. Strikingly, deamidated RelA binds DNA in vitro and demonstrates the ability to up-regulate key glycolytic enzymes, thus promoting aerobic glycolysis, which provides metabolites for de novo nucleotide synthesis [71]. Genetic depletion and pharmacological inhibition of CAD effectively deplete the nucleotide pool and boost the antiviral inflammatory response in cells infected with SARS-CoV-2 [71]. Particularly, 2-TCPA, a glutamine analog, potently inhibits CAD in vitro and in cells, thereby reducing SARS-CoV-2 replication ex vivo and in mouse models. These results highlight the feasibility of targeting a host metabolic enzyme for antiviral therapeutic applications. Though how viruses interact with dihydroorotate dehydrogenase (DHODH) remains unknown, multiple inhibitors of DHODH show antiviral activity against a broad spectrum of viral pathogens, including influenza A virus, Zika virus, Ebola virus, and SARS-CoV-2 [72,73]. SARS-CoV-2 ORF7b and ORF8 activate CTP synthetase 1 (CTPS1) to promote de novo CTP synthesis, while shutting down interferon production [74]. The enzyme IMP dehydrogenase (IMPDH) catalyzes an essential step in the de novo biosynthesis of guanine nucleotides, namely, the conversion of IMP to XMP. IMPDH inhibitors also show a broad-spectrum antiviral activity, such as HBV, HCMV, respiratory syncytial virus (RSV), HSV-1, parainfluenza-3 virus, encephalomyocarditis virus (EMCV), and Venezuelan equine encephalomyelitis virus (VEEV). [75]. Aspartate is an essential precursor of nucleotide synthesis. Inhibition of argininosuccinate synthetase 1 (AS1) increases the availability of its substrate, aspartate, thereby promoting both pyrimidine and purine synthesis. Likewise, down-regulation of AS1 enhances the genome replication and virion production of HSV-1 [76]. Some viruses stimulate nucleotide synthesis via expressing their own metabolic enzymes. For instance, HSV-1 encodes thymidine kinase, ribonucleotide reductase, dUTPase, and uracil-DNA glycosylase, which catalyze the bottle-necked steps of nucleotide synthesis, thus collectively contributing to an elevated nucleotide pool [77,78].

#### 2.2.4. Lipogenic Enzymes

**Figure 5:**
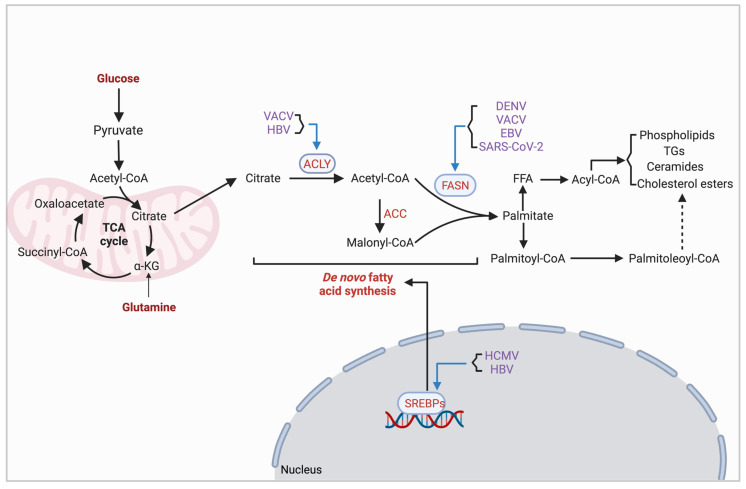
Lipogenic enzymes are hijacked by viruses. Figure shows a schematic illustration of the cross-regulation between viruses and lipogenic enzymes. Abbreviations: α-KG, α-ketoglutarate; ACLY, ATP citrate lyase; VACV, vaccinia virus; HBV, hepatitis B virus; ACC, acetyl-CoA carboxylase; FASN, fatty acid synthase; DENV, dengue viruses; EBV, Epstein–Barr virus; SARS-CoV-2, severe acute respiratory syndrome coronavirus 2; FFA, free fatty acid; TGs, triglycerides; SREBPs, sterol regulatory element binding proteins; HCMV, human cytomegalovirus. Created with Biorender.com.

Host lipids are crucial for viral infection by providing essential components to complete key processes. As such, viruses often activate lipid synthesis pathways to increase the supply (Figure 5) (Table 1). Sterol regulatory element binding proteins (SREBPs) are the principal regulators that control cellular lipid levels. HCMV infection induces protein kinase R(PKR)-like endoplasmic reticulum (ER) kinase (PERK) expression, which stimulates SREBP1 cleavage and the activation of lipogenesis [79,80]. HCMV infection also increases the expression of diverse enzymes involved in the synthesis of very-long-chain fatty acids (VLCFAs), such as acyl-CoA synthetases and elongases. Drugs that inhibit the synthesis of VLCFAs reduce the infectivity of HCMV progeny virions [81]. DENV infection stimulates fatty acid biosynthesis, and the de novo-synthesized lipids are incorporated into sites of DENV replication. Nonstructural protein 3 (NS3) of DENV recruits fatty acid synthase (FASN) to sites of DENV particle replication and stimulates FASN activity. Cerulenin and C75, which are FASN inhibitors, significantly reduce DENV replication [82]. Likewise, the suppression of FASN through C75 and acetyl-CoA carboxylase (ACC) via TOFA in VACV-infected cells greatly diminished the viral yield, both of which can be partially rescued by exogenous palmitate, the predominant saturated fatty acid [83]. Cerulinin also significantly inhibits the glycoprotein maturation and replication of varicella zoster virus (VZV) [84]. Transgenic mice with the HBV pre-S2 mutant antigen exhibit increased accumulation of lipid droplets. It appears that HBV pre-S2 activates the sterol regulatory element binding transcription factor 1 (SREBF1) to up-regulate ACLY, which then activates fatty acid desaturase 2 (FADS2), mediated through ACLY-dependent histone acetylation [85]. EBV infection alters lipid metabolism partially through EBV-encoded RNAs (EBERs), which are capable of increasing the expression of FASN and LDLR [86]. Quercetin, known to inhibit FASN, was found to inhibit the proliferation of NPC cells [86]. Another component of EBV, the immediate–early protein BRLF1, induces the up-regulation of FASN during lytic replication [87]. Orlistat, a FASN inhibitor, can potently diminish SARS-CoV-2 replication both in vitro and in vivo [44], indicating the essential role of lipid synthesis in SARS-CoV-2 replication.

## 3. Metabolic Enzymes and Innate Immunity

### 3.1. Metabolic Enzymes and the Interferon Induction Pathway

**Figure 6:**
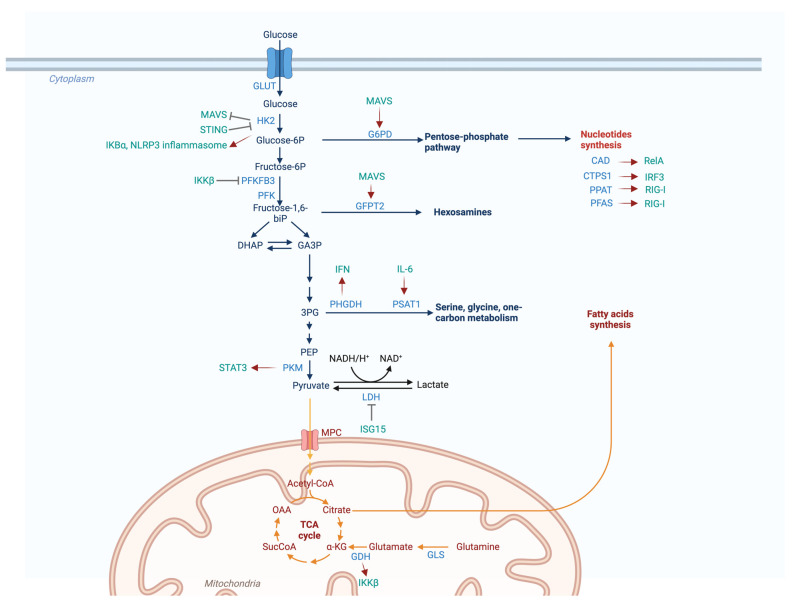
Metabolic enzymes interact with innate immunity. Figure shows a schematic illustration of the cross-regulation between innate immune modulators and metabolic enzymes in various pathways, including glycolysis, the TCA cycle, and nucleotide synthesis. Abbreviations: GLUT, glucose transporter; HK2, hexokinase-2; MAVS, mitochondrial antiviral-signaling protein; STING, stimulator of interferon gene; IκBα, inhibitor of NF-κB α; PFKFB3, 6-phosphofructo-2-kinase/fructose-2,6-biphosphatase 3; PFK, phosphofructokinase; IKKβ, inhibitor of nuclear factor kappa-B kinase subunit beta; PKM, pyruvate kinase muscle isozyme; STAT3, signal transducer and activator of transcription 3; LDH, lactate dehydrogenase; ISG15, interferon stimulated gene 15; G6PD, glucose-6-phosphate dehydrogenase; GFPT2, glutamine-fructose-6-phosphate transaminase 2; PHGDH, phosphoglycerate dehydrogenase; PSAT1, phosphoserine aminotransferase 1; IFN, interferon; IL-6, interleukin 6; GDH, glutamate dehydrogenase; GLS, glutaminase; CAD, carbamoyl-phosphate synthetase, aspartate transcarbamoylase and dihydroorotase; CTPS1, cytidine triphosphate synthase 1; PPAT, phosphoribosyl pyrophosphate amido transferase; PFAS, phosphoribosylformylglycinamidine synthase; IRF3, interferon regulatory factor 3; RIG-I, retinoic acid-inducible gene-I; DHAP, dihydroxyacetone phosphate; GA3P, glyceraldehyde 3-phosphate; PEP, phosphoenolpyruvic acid; α-KG, α-ketoglutarate; SucCoA, Succinyl-CoA; OAA, oxaloacetate. Created with Biorender.com.

Reciprocal interaction between the interferon induction pathway and metabolism is emerging as a topic of investigation (Figure 6). Mitochondrial antiviral signaling protein (MAVS) is inhibited by the glycolytic product lactate and glycolytic enzyme HK2, thus suppressing the innate immune activation downstream of cytosolic double-stranded (ds)RNA [88,89]. The stimulator of interferon gene (STING) is an ER-resident protein and requires sulfate glycosaminoglycans (sGAG), which serve as the ligands of STING in mediating IFN induction by cytosolic DNA [90,91,92]. sGAG-associated metabolic enzymes and transporters are thus engaged in the cGAS-dependent DNA-sensing pathway. The antiviral activity of MAVS and interferon regulatory factor 5 (IRF5) is favored by O-GlcNacylation [93,94], which requires an active hexosamine biosynthesis pathway to sustain the production of sugar donors. Phosphoglycerate dehydrogenase (PHGDH) depletion increases the expression of V-ATPase subunit ATP6V0d2, which can induce the lysosomal degradation of YAP, the Hippo pathway transcriptional co-activator that is capable of disrupting the TBK1-IRF3 interaction, concomitantly inhibiting interferon induction [95]. Furthermore, accumulating studies collectively support the conclusion that interferon signaling can regulate metabolism to tune the host immune defense against microbial infection. The depletion of interferon receptors significantly boosts aerobic glycolysis in usutu virus (USUV)-infected cells [96]. Interferon treatment can restrain glycolysis in macrophages infected with *M. tuberculosis* [97] and epithelial cells infected with *C. trachomatis* [98]. Counterintuitively, IFN-β was also shown to induce glycolysis in mouse embryo fibroblasts, which is crucial for the acute antiviral response against coxsackievirus B3 infection [99]. Moreover, multiple components of the interferon signaling pathway directly modulate metabolic enzymes to differentially reprogram metabolism. STING can bind to HK2 and inhibit its enzymatic activity as well as glycolysis [100]. He et al. revealed that MAVS interacts with the G6PD and glutamine-fructose-6-phosphate transaminase (GFPT2) [101]. As such, MAVS can divert the carbon flux from glycolysis to the pentose phosphate pathway (PPP) and hexosamine biosynthesis pathway upon the activation of the RNA-sensing pathway. The IFN-inducible ubiquitin-like molecule interferon stimulated gene-15 (ISG15) can be covalently conjugated to a series of glycolytic enzymes such as lactate dehydrogenase A (LDHA), thereby repressing their enzymatic activity [102].

In addition to the post-translational regulation, the interferon signaling pathway directly induces the expression of an array of metabolic enzymes. Some of these enzymes execute antiviral function by orchestrating a catabolic program that prevents viruses from utilizing host metabolites. For instance, cholesterol-25-hydroxylase (CH25-H) converts cholesterol into 25-hydroxycholesterol (25-HC). Given the importance of cholesterol in cellular membrane, CH25-H impedes the progression of the virus life cycle at multiple stages, ranging from entry to budding [8,103,104]. Sterile α-motif and histidine-aspartic acid domain-containing protein 1 (SAMHD1) act as a deoxynucleotide triphosphate (dNTP) hydrolase that degrades and depletes dNTPs, resulting in the exhaustion of the deoxyl nucleotide pool to restrict DNA synthesis, which is critical for retrovirus integration [105]. Another interferon-stimulated gene (ISG), viperin, can covert cellular cytidine 5′-triphosphate (CTP) into 3′-deoxy-3′,4′-didehydro-CTP (ddhCTP), which causes chain termination when incorporated into a newly synthesized viral RNA [106]. Indoleamine-2,3-dioxygenase (IDO1) mediates a broad-spectrum antiviral effect by dictating the catabolism of tryptophan, an essential amino acid for viral replication [107]. However, long-term tryptophan exhaustion can lead to immunosuppression, which may be beneficial for viral infection, particularly persistent infection [108]. Polyamine deprivation comprises another ISG-mediated antiviral strategy. Spermidine/spermine N1-acetyltransferase 1 (SAT1) can impair the replication of multiple RNA viruses, such as Chikungunya virus and Zika virus, by promoting polyamine catabolism to deplete the polyamine supply essential for viral replication [109,110,111].

Several interferon-inducible metabolic enzymes are capable of mediating the post-translational modification of viral proteins. For example, poly (ADP-ribose) polymerases (PARPs) have been characterized as ISGs [112,113]. By adding mono-ADP-ribose to target proteins, PARPs can function as an antiviral effector through modifying viral proteins [114]. This post-translational modification requires NAD^+^ to serve as the donor of ADP-ribose, and up-regulation of PARPs can exhaust the cellular NAD^+^ pool [115]. Interferon signaling can directly drive the expression of nicotinamide phosphoribosyltransferase (NAMPT), the rate-limiting enzyme of the NAD^+^ salvage synthesis pathway [116,117,118]. The interferon-mediated induction of NAMPT may support the antiviral response, at least partially, through replenishing the cellular NAD^+^ pool to fuel ADP-ribosylation. Additionally, interferon signaling induces nitrogen monoxide synthetase type-2 (NOS2/iNOS) expression [119]. iNOS can catalyze NO production, which promotes the S-nitrosylation of viral proteins, thereby exerting an antiviral effect [120].

### 3.2. Metabolic Enzymes and the Inflammatory Pathway

Inflammation has been recognized to shape the cellular metabolic landscape [121]. These effects underlie the reciprocal and tight regulation between inflammatory pathways and metabolic enzymes (Figure 6). A number of metabolic enzymes were shown to function as “moonlighting enzymes” that can facilitate protein post-translational modification apart from their metabolic activity [122]. HK2 can act as a protein kinase that phosphorylates inhibitor of NF-κB α (IκBα) and induces its degradation to trigger NF-κB activation [123]. PKM2 can phosphorylate signal transducer and activator of transcription-3 (STAT3), a versatile transcriptional factor downstream of multiple cytokine signaling pathways, and promote its transcriptional activity [124]. Intriguingly, HK2 also acts as a pattern-recognition receptor that detects bacterial peptidoglycan in the cytoplasm and subsequently activates the NLRP3 inflammasome [125]. Fructose-2,6-bisphosphatase TIGAR (Tp53-induced glycolysis and apoptosis regulator) can suppress NF-κB activation by disassociating NEMO from the ubiquitination complex LUBAC [126]. Moreover, some metabolic enzymes can regulate inflammation through producing certain immunometabolites. Multiple glycolytic enzymes can contribute to the NLRP3 inflammasome activation by undermining aerobic glycolysis [127,128]. As a key metabolite of the TCA cycle, α-KG can directly activate the canonical NF-κB kinase IKKβ [129]. Another TCA cycle intermediate, succinate, is well-known for its pro-inflammatory effect through activating NLRP3 in LPS-primed macrophages [130], while itaconate, a derivative of aconitate, possesses anti-inflammatory activity [131]. Itaconate can modify NLRP3 through dicarboxypropylation, thereby inhibiting the inflammatory activity of the NLRP3 inflammasome [132]. Fumarate can suppress gasdermin D (GSDMD)-induced pyroptosis by mediating its succinylation [133]. Consequently, the immunomodulatory activities of metabolites convey an indirection on inflammation, reflecting their corresponding enzymes in the inflammatory response. Glutamate dehydrogenase 1 (GDH1) binds to IKKβ and generates α-KG from glutamate to facilitate IKKβ activation and the inflammatory response [129]. Aconitate decarboxylase-1 (ACOD1) negatively regulates inflammation by modulating the conversion of aconitate to itaconate [131].

Inflammatory pathways also manifest profound regulatory effects on metabolic enzymes (Figure 6). IKKβ can directly phosphorylate and suppress the glycolytic enzyme 6-phosphofructo-2-kinase/fructose-2, 6-bisphosphatase (PFKFB3) [134]. Depletion of PFKFB3 abrogates TNF-α-induced NF-κB activation in endothelial cells, suggesting a potential feedback loop [134]. At the transcriptional level, multiple metabolic genes have been characterized as direct targets of the canonical NF-κB pathway, covering glycolysis, oxidative phosphorylation, and the TCA cycle [135,136,137]. In mouse embryo fibroblasts, RelA/p65 is capable of inhibiting glycolytic gene expression while promoting oxidative phosphorylation gene expression in a p53-dependent manner [137]. Meanwhile, in p53-deficient cancer cells, RelA/p65 can be shuttled to the mitochondria, where it represses oxidative respiration-associated gene transcription [138,139]. Moreover, RelA-mediated glycolytic gene expression can augment the well-known Warburg effect in cancer cells [140,141,142]. RelA is also crucial for maintaining beta cell function by governing the expression of a metabolic gene network [143]. Remels et al. [144] reported that cytokine-induced NF-κB activation impairs the expression of oxidative phosphorylation genes in cardiomyocytes, which is possibly attributed to PGC1 (PPARγ coactivator 1) inhibition. NF-κB and STAT3 can cooperate with HIF-1α to regulate metabolic gene expression. They are capable of either inducing HIF1A gene transcription or acting as co-factors of HIF-1α to up-regulate glycolytic gene transcription [141,145,146]. Taken together, these studies collectively support the conclusion that the NF-κB pathway regulates energy metabolism in a highly context-dependent manner. In parallel to enhanced aerobic glycolysis, dampened mitochondrial oxidative activity is also observed in cells expressing constitutively activated STAT3 [147]. Furthermore, STAT3 inhibits the transcription of gluconeogenic genes (i.e., G6Pase, PEPCK) through binding to their promoter region [148]. This process may, at least partially, account for how cytokines regulate liver insulin sensitivity [149]. STAT3 can also undergo mitochondrial translocation, which is instigated by the phosphorylation of serine 727 residue [150]. Mitochondrial STAT3 can potentiate the electron transport chain by enhancing complexes I and II and ATP synthetase activity [151]. In agreement with its role as an oncogenic protein, mitochondrial STAT3 favors the growth of multiple cancer cell lines [151,152] and Ras-mediated cellular transformation [153]. Wang et al. reported that in TSC2-deficient cells, IL-6 can provoke serine synthesis by up-regulating the expression of phosphoserine aminotransferase 1 (PSAT1) in a STAT3-independent manner [154].

### 3.3. Viral Enzymes Mute Host Immune Response via Cellular Metabolic Enzymes

Furthermore, some virus-encoded metabolic enzymes can impede the host antiviral response via targeting key signaling molecules of the immune system. For example, UL50 proteins encoded by pseudorabies virus (PRV) and HSV-1 impede type I IFN-induced STAT1 phosphorylation by accelerating the lysosomal degradation of IFN receptor 1 (IFNAR1) [155]. Interestingly, murine gamma herpesvirus 68 and KSHV encode homologs of phosphoribosylformylglycinamidine synthetase (PFAS), a scaffold crucial for the assembly of the purinosome responsible for de novo purine synthesis, to negate dsRNA-induced innate immune activation [156]. PFAS belongs to the glutamine amidotransferase (GAT) family, which catalyzes the synthesis of nucleotides, amino acids, glycoproteins, and an enzyme cofactor, NAD^+^ [157]. Unlike PFAS, these gamma herpesvirus homologues lack the key residues required for enzyme catalysis, and thus are designated viral pseudoenzymes or vGATs [157]. Mechanistically, vGAT proteins recruit cellular PFAS to deamidate and activate RIG-I, which enables the RTA-mediated degradation of RelA and thus, the blockade of antiviral cytokine production [156]. In contrast, HSV-1 UL37 acts as a bona fide enzyme that deamidates RIG-I and cGAS, thereby muting the innate immune activation by dsRNA and dsDNA [158,159]. In deamidating RIG-I, the sequential deamidation events of N495 and N549 are required to derive the dsRNA-binding activity of RIG-I [158]. UL37-mediated deamidation of N495 enables the binding of RIG-I to the pyrophosphate amidotransferase (PPAT) and subsequent deamidation of N549 [160]. This study reveals an intricate cooperation between a viral deamidation and cellular GAT in evading RIG-I-mediated innate immune activation. Taken together, cellular GAT enzymes may constitute key signaling nodes that integrate communication between cellular metabolism and the antiviral immune response.

## 4. Targeting Metabolic Enzymes as an Antiviral Strategy

Targeting host factors represents a more broad-spectrum antiviral approach. As obligate intracellular pathogens, viruses are entirely dependent on host metabolites. Thus, host metabolic enzymes can be potential targets for antiviral drug development.

Nucleotide synthesis consists of the de novo and salvage synthesis pathways. Viruses usually activate de novo nucleotide synthesis to satisfy their excessive demand for nucleotides [161]. Therefore, de novo nucleotide synthesis can be a potential therapeutic target. The first and rate-limiting enzyme in de novo pyrimidine synthesis, CAD, has been characterized as a host factor crucial for the infection of a number of viruses. The large, trifunctional CAD enzyme contains carbamoyl-phosphate synthetase, aspartate transcarbamoylase, and dihydroorotase, which are coordinated by an intramolecular tunnel to deliver intermediates of de novo pyrimidine synthesis. The development of a CAD inhibitor was originally reported in the 1970s. The initial CAD inhibitor N-phosphonoacetyl-L-aspartate (PALA) targets the aspartyl transcarbamoylase domain, which catalyzes the second step of de novo pyrimidine synthesis [162]. Several clinical trials using PALA to treat cancers were carried out but failed due to limited anti-tumor activity or excessive toxicity [163], while the antiviral activity of CAD inhibitors was poorly investigated. Our lab reported a novel glutamine analog, 2-TCPA, which specifically inhibits CAD and potently reduces SARS-CoV-2 replication [71]. Consistent with the importance of CAD in SARS-CoV-2 infection, SARS-CoV-2 potently activates CAD to boost the nucleotide supply while shutting down NF-κB activation and antiviral cytokine production. To do that, CAD deamidates RelA, which shunts RelA from mediating an inflammatory response to aerobic glycolysis. Mechanistically, deamidated RelA failed to activate the transcription of known NF-κB-responsive promoters, instead activating that of diverse glycolytic genes [164]. By blocking SARS-CoV-2-induced CAD activation, 2-TCPA inhibits both de novo pyrimidine synthesis and RelA deamidation, consequently boosting inflammatory cytokine production and inhibiting nucleotide synthesis. This agent exhibited considerable anti-SARS-CoV-2 activity in cell lines and mouse models. However, the antiviral effects of CAD inhibitors on other viruses are waiting for further exploration. Catalyzing a step of de novo pyrimidine synthesis immediately downstream of CAD, dihydroorotate dehydrogenase (DHODH) serves as a key enzyme coupling pyrimidine synthesis to oxidative phosphorylation, which transfers the electron generated from DHO oxidation to co-enzyme Q/ubiquinone [165]. DHODH inhibitors exert antiviral effects against a broad spectrum of viral pathogens, including coronavirus, influenza virus, flavivirus, Ebola virus, and HSV-1, in cultured cells [72,166,167,168,169,170]. Interestingly, inhibiting DHODH not only blocks de novo pyrimidine synthesis, but also augments interferon response against viral infection [169,171]. Lucas-Horani et al. reported that the increase in transcription of ISGs elicited by DHODH inhibitors was completely abrogated by the addition of uridine [172], suggesting that the nucleotide pool imbalance may account for how DHODH inhibitors evoke the interferon response. Moreover, supplementation of uridine can completely diminish the antiviral effect of the DHODH inhibitor brequinar [173,174,175], implicating that the antiviral effect of DHODH inhibition is mainly dependent on pyrimidine synthesis rather than mitochondrial oxidative phosphorylation. Although the DHODH inhibitors leflunomide and teriflunomide are clinically available, they are currently only approved for autoimmune disease treatment [176], while other application in treating viral infection is still under investigation.

De novo purine synthesis can also be targeted to block viral replication. Inhibition of inosine 5′-monophosphate dehydrogenase (IMPDH) impairs the synthesis of guanine nucleotides, leading to the exhaustion of GTP [177]. A clinically available IMPDH inhibitor, ribavirin, has been approved to treat the infection of hepatitis C, RSV, and several kinds of viral hemorrhagic fevers [178]. As a guanosine analog, ribavirin establishes antiviral effects through not only IMPDH inhibition, but also blockade of viral RNA synthesis [178]. IMPDH inhibition-induced GTP deprivation can further increase the frequency of ribavirin incorporation into viral RNA, forming a positive feedback loop to aggravate its antiviral effect [179].

Inhibition of glycolysis by the glucose analog 2-deoxy-D-glucose (2-DG) manifests a favorable and broad-spectrum antiviral effect in cell lines [180]. However, animal studies showed that the application of 2-DG in vivo may bring complicated outcomes in HSV-1 infection. Administration of 2-DG at an early stage of HSV-1 infection in eyes can aggravate viral dissemination and result in lethality [181]. Meanwhile, two early studies showed that 2-DG has no effect on HSV-1 and HSV-2 cutaneous infection in mice and guinea pigs [182,183]. These observations suggest that the effect of inhibitors of metabolic enzymes on viral infection and diseases thereof must be considered in the context of the host immune response.

Inhibition of FASN is another strategy to treat viral infection. FASN is required for de novo fatty acid synthesis by catalyzing the synthesis of palmitate, which favors the viral life cycle by supplying lipid synthesis and fueling viral protein palmitoylation [83]. Pharmacological inhibition of FASN shows a broad-spectrum antiviral activity against enveloped viruses such as SARS-CoV-2 [44,82,86,184]. An FDA-approved anti-obesity drug, orlistat, can effectively suppress SARS-CoV-2 replication in cells and in mouse models. Hence, targeting FASN for inhibition can be a potential strategy to treat viral infection, especially enveloped viruses, which are more dependent on lipids.

## 5. Discussion

In the current review, we have consolidated recent discoveries regarding the involvement of metabolic enzymes in viral infections and hosts’ immune responses. Specifically, our primary focus revolves around metabolic enzymes engaged in glycolysis, the TCA cycle, nucleotide synthesis, and lipogenesis.

As obligate intracellular pathogens, it is not surprising that viruses hijack cellular metabolic enzymes to facilitate their replication. Viruses utilize metabolic enzymes to specifically facilitate some stage of the viral life cycle (e.g., metabolic receptors for viral entry) or generate essential biomolecules for rapid replication. It is worth mentioning that some metabolic enzymes may function as antiviral factors, such as cholesterol-25-hydroxylase and PFAS. Nevertheless, there is a scarcity of studies on this subject. Delving into the specific role of each metabolic enzyme in different viruses is crucial for designing antiviral strategies that target these enzymes.

Numerous studies have unveiled the diverse strategies employed by viruses to regulate the expression or activities of metabolic enzymes. In addition, these studies have uncovered potential targets for the development of antiviral drugs. However, metabolic enzymes play a crucial role in immune cells, especially lymphocytes. Inhibiting the metabolic enzymes activated by the virus may consequently result in a constrained immune response. Delving into the distinct roles of metabolic enzymes in viral infection and the immune response promises to offer fresh insights into potential antiviral targets.

The interaction between metabolism and immune response is intricate, with metabolic enzymes playing a pivotal role. Continued research into the crosstalk between metabolic enzymes and the immune response promises to unveil the significance of immunometabolism in viral infections and cancers. Furthermore, metabolic enzymes can catalyze protein post-translational modifications. Examining the impact of these modifications on viral replication and immune response not only presents an intriguing topic but also holds practical significance for antiviral development.

## Figures and Tables

**Table 1:** Metabolic alteration in various virus infections.

Metabolic Pathways	Metabolic Enzymes/Regulators/Pathways	Viruses	Effects	Virus Types
Glycolysis	HK2, PFKP	AdV	Up-regulating expression	DNA virus
	PFK-1	HSV-1	Up-regulating expression	DNA virus
	PFK, Pyruvate kinase, GLUT4	HCMV	Up-regulating expression	DNA virus
	PFK		Up-regulating enzymatic activity	
	GLUT1		Down-regulating expression	
	HK2, PDK1, GLUT1, PKM2	EBV	Up-regulating expression	DNA virus
	PKM2	KSHV	Up-regulating expression	DNA virus
	GLUT1, HK2	DENV	Up-regulating expression	RNA virus
	PKM2	HPV	Down-regulating enzymatic activity	DNA virus
	GLUT1	HBV	Up-regulating expression	DNA virus
	HK2	HCV	Up-regulating enzymatic activity	RNA virus
	HIF-1α	SARS-CoV-2	Up-regulating transcriptional factor activity	RNA virus
	HK2, PFKM, PKM2	CVB3	Up-regulating expression	RNA virus
Pentose phosphate pathway	G6PD	HBV	Up-regulating expression	DNA virus
TCA cycle	Glutamine anaplerosis, reductive carboxylation	AdV	Activating the pathways	DNA virus
	SLC1A5	KSHV	Up-regulating expression	DNA virus
	GLS, GDH	HCMV	Up-regulating expression and enzymatic activity	DNA virus
	GLS	HCV	Up-regulating expression	RNA virus
	PC	SARS-CoV-2	Up-regulating expression	RNA virus
	Oxidative glutamine metabolism		Inhibiting pathway activity	
	Reductive carboxylation		Activating pathway activity	
Nucleotide synthesis	CAD	SARS-CoV-2	Up-regulating enzymatic activity	RNA virus
	CTPS1			
	Viral nucleotide synthetase	HSV-1	Enhancing host nucleotide synthesis	DNA virus
Lipid synthesis	SREBP1	HCMV	Up-regulating transcriptional factor activity	DNA virus
	VLCFA synthetic enzymes		Up-regulating expression	
	FASN	DENV	Up-regulating enzymatic activity	RNA virus
	SREBF1	HBV	Up-regulating transcriptional factor activity	DNA virus
	FASN, LDLR	EBV	Up-regulating expression	DNA virus

## Data Availability

Not applicable.
